# Native and Non-Native Soil and Endophytic *Trichoderma* spp. from Semi-Arid Sisal Fields of Brazil Are Potential Biocontrol Agents for Sisal Bole Rot Disease

**DOI:** 10.3390/jof10120860

**Published:** 2024-12-11

**Authors:** Leonardo O. Barbosa, Tainá D. S. da Conceição, Adriana de O. Neves, Wélica Z. B. Rocha, Beatriz S. Damasceno, Paula L. C. Fonseca, Paulo R. Ribeiro, Luis M. R. Tome, Dener E. Bortolini, Fabiano M. Martins, Fábio T. Raya, Aristóteles Goes-Neto, Ana C. F. Soares

**Affiliations:** 1 Center of Agricultural, Environmental and Biological Sciences, Federal University of Recôncavo of Bahia (UFRB), Cruz das Almas 44380-000, BA, Brazil; barbosleonard@gmail.com (L.O.B.); taina.delmondes@outlook.com (T.D.S.d.C.); agrooneves@gmail.com (A.d.O.N.); welicazbr@usp.br (W.Z.B.R.); beatriz.damasceno@ufv.br (B.S.D.); fmartins@ufrb.edu.br (F.M.M.); 2 JCO Bioprodutos Company, Barreiras 47810-423, BA, Brazil; 3 Department of Phytopathology and Nematology, Escola Superior de Agricultura Luiz de Queiroz, Universidade de São Paulo, Piracicaba 13418-260, SP, Brazil; 4 Departament of Phytopathology, Federal University of Viçosa, Vicosa 36570-900, MG, Brazil; 5 Laboratory of Molecular and Computational Biology of Fungi (LBMCF), Institute of Biological Sciences, Universidade Federal de Minas Gerais (UFMG), Belo Horizonte 31270-901, MG, Brazil; camargos.paulaluize@gmail.com (P.L.C.F.); lmarcelotome@gmail.com (L.M.R.T.); gigatonn@gmail.com (D.E.B.); arigoesneto@icb.ufmg.br (A.G.-N.); 6 Institute of Chemistry, Department of Organic Chemistry, Federal University of Bahia, Salvador 40110-909, BA, Brazil; paulodc3@gmail.com; 7 Laboratory of Genomics and BioEnergy (LGE), Institute of Biology, University of Campinas (Unicamp), Campinas 13083-970, SP, Brazil; raya@unicamp.br

**Keywords:** *Agave sisalana*, *Aspergillus welwitschiae*, *Trichoderma* cf. *asperellum*, *Trichoderma harzianum* (species complex)

## Abstract

Sisal (*Agave sisalana*) bole rot caused by *Aspergillus welwitschiae* is the main phytosanitary problem affecting sisal in the Brazilian semi-arid region. The aim of this study was to evaluate *Trichoderma* spp. as biocontrol agents for sisal bole rot. Native and non-native species, both soil inhabitants and endophytes, and isolated from different plant hosts were tested. Anatomical studies of the interaction among *A. sisalana*, *Trichoderma* spp., and *A. welwitschiae* were performed. *T.* cf. *asperellum* (isolate F12), an endophyte of sisal leaves; *T.* cf. *asperellum* (TCS83) from banana plant soil; *T. lentiforme* (TCS15) and *T. harzianum* (species complex) (TCS35 and TCS76) from sisal root soil; *T. spirale* (R62) and *T. saturnisporum* (R75), endophytes of sisal roots, were the most efficient isolates, with inhibition of *A. welwitschiae* mycelial growth by up to 70%, and inhibition of sporulation and spore germination by 99%. A reduction in disease incidence of 70 to 93% and in disease severity of 97% was achieved. *T. lentiforme* (TCS1), *T. harzianum* (species complex) (TCS35 and R72), and *T. koningiopsis* (R78) showed mycoparasitism. An increase in cell wall thickness of bole tissue colonized by these *Trichoderma* species indicated that induced plant defense responses occurred, preventing pathogen colonization, which should be further investigated. Native and non-native *Trichoderma* species can control sisal bole rot disease.

## 1. Introduction

Agaves have been cultivated for thousands of years for their applications in food, beverages, and fiber production. Their fibers, among the most important natural hard fibers, are widely used in items such as bailey twigs, cords, and handcrafts [1,2,3,4]. Agaves are endemic to the American continent and are distinguished by their resilience, which allows them to withstand drought, high temperatures, and intense solar radiation due to their Crassulacean Acid Metabolism (CAM) [1,5,6]. Given the current challenges posed by climate change and its impact on agriculture, Agave species are attracting renewed interest as feedstock for bioenergy and other uses, particularly in regions with challenging environmental conditions for conventional agriculture [7,8].

In Brazil, *Agave sisalana* known as sisal, was introduced in 1903, and it has been cultivated for fiber extraction since then, but with a greater and dominant production in the Northeastern part of Brazil [9,10]. In fact, since the 1960s, Brazil has been the largest worldwide sisal fiber producer, with approximately 40% of the world’s production [11], and 94% of Brazil´s production is concentrated in the semi-arid region of Bahia state [12]. Sisal fiber is also produced in countries such as Angola, Kenya, Madagascar, Mozambique, South Africa, Tanzania, China, Indonesia, Thailand, Cuba, and Haiti [11]. However, there has been a decrease in sisal fiber production in Brazil, with a reduction of over 50% in its production in the last two decades [11]. Factors such as long drought periods, low investment in research and technology, and sisal bole rot disease have been pointed out as the main reasons for this production decline [10,13].

Sisal bole rot disease has been the main phytosanitary problem for several decades in Brazil, threatening the sustainability of fiber production in this country [14,15]. Primary symptoms include bole and leaf base rot, characterized by reddish to dark brown discoloration of the internal tissue, while secondary symptoms include leaf yellowing and wilting. With disease progression, sisal bole becomes completely rotten, causing plant death [14]. This disease can manifest at various stages of plant development, and its causal agent is the saprotrophic fungus *Aspergillus welwitschiae*, which transitions to a necrotrophic lifestyle when invading injured sisal boles [14].

In Brazil, sisal fiber production is predominantly managed by small family-owned farms. The farmers typically have limited investments in soil and water management practices, as well as agricultural inputs. Additionally, there is a notable absence of selected sisal varieties and the use of nursery plants with established genetic, nutritional, or phytosanitary standards [15]. These circumstances have contributed to the dissemination and increase in bole rot disease in all sisal-producing areas [10,15]. Recommended control methods for this disease are based on the removal of symptomatic plants from the field and the visual selection of healthy plant suckers for planting, but very few farmers follow these recommendations [10]. Furthermore, a 78 to 88% pathogen incidence in sisal plants was shown in all tested sisal-producing areas of Bahia state through molecular epidemiology techniques [15]. According to these authors, contaminated plant suckers, used by sisal farmers as planting materials, are the major source of disease dissemination. Therefore, the development of methods for controlling bole rot disease is a priority for sisal sustainable management.

Given that *A. welwitschiae* is a ubiquitous, saprotrophic, endophytic, and opportunistic fungus spreading via soil, air, and plant material [16,17], biological control is a promising approach for managing sisal bole rot. In fact, biological control has exhibited promising outcomes against soil-borne pathogens capable of surviving in plant residues and generating resilient spores like chlamydospores [18]. It stands as a preferred approach alongside genetic control methods in combating these pathogens. Studies on the bioprospection of biological control agents (BCAs) for sisal bole rot control have been reported, mostly with rhizobacteria and endophytic bacteria [19,20,21] and with fungi such as *Penicillium citrinum* [22].

The ubiquitous fungus *Trichoderma* (Teleomorph: Hypocrea) is amongst the most studied BCAs of plant pathogens, especially with soil-borne pathogenic fungi [18,23]. Species of this fungus are generally soil inhabitants and saprophytes, which can also occur as opportunistic and avirulent plant symbionts [24,25]. Numerous *Trichoderma* species have the capacity to effectively diminish both disease occurrence and intensity through several mechanisms, such as the production of enzymes that degrade fungal cell walls, secondary metabolites with antimicrobial activities, mycoparasitism, induction of plant defense responses, and competition for resources and space in the rhizosphere or within plant tissues as endophytic fungi [24,25,26,27,28,29]. For instance, *T. harzianum*, strain ITEM 3636, was described as a biofungicide for controlling peanut brown root rot caused by *Fusarium solani*, with the synthesis of secondary metabolites and enzymes such as chitinases, proteases, N-acetyl-β-D-glucosaminidases, and glucanases, with alterations in pathogen’s hyphae [30]. For *Agave* plants, studies with *Trichoderma* are scarce and mostly with *A. tequilana*. For instance, T. *harzianum*, *T. virens,* and *T. aureoviride* were tested for control of *A. tequilana* wilt caused by species of *Fusarium* and *Thielaviopsis paradoxa*, and the authors showed that these species of *Trichoderma* could colonize the soil and plant roots and control the pathogens population in soil [31]. *Trichoderma longibrachiatum* was also shown to inhibit the growth of *T. paradoxa* from *A. tequilana* [32].

Although most studies have tested *Trichoderma* isolates from the same environment or host plant, these fungi can adapt to different environmental conditions and can control several plant fungal pathogens [25]. Indeed, our research group has had positive results on disease control with *Trichoderma* isolated from different plants and biomes. Therefore, in our work, we have tested isolates from sisal plantations in Bahia state, as well as from other crops and environments.

This study aimed to i) evaluate the antagonistic effects of both native and non-native *Trichoderma* species against *A. welwitschiae*; ii) determine the efficacy of *Trichoderma* spp. in controlling sisal bole rot; and iii) describe the anatomical interactions among *Trichoderma*, *A. welwitschiae*, and *A. sisalana* within bole tissue.

## 2. Materials and Methods

### 2.1. Molecular Identification of Trichoderma spp. and A. welwitschiae Isolates

Eighteen isolates of *Trichoderma* spp. from various ecological niches and one isolate of *A. welwitschiae* (A1P1) from the Laboratory of Agricultural Microbiology, Federal University of Recôncavo of Bahia, were used in this study (Table 1). The isolates were cultured on malt extract agar (MEA) at 28 ± 2 °C for 5 to 10 days. DNA was extracted using the FastDNA Spin Kit (MP Biomedicals), following the manufacturer’s protocol, and purified with the High Pure PCR Template Preparation Kit (Roche). DNA quality and quantity were assessed by agarose gel electrophoresis and spectrophotometry using the Nanodrop 100 ND (Thermo Scientific, Madison, WI, USA).

Amplifications targeted three nuclear regions. The first was the internal transcribed spacer (ITS), widely recognized as the primary fungal barcode [33], amplified with primers ITS 4 (5′-TCCTCCGCTTATTGATATGC-3′) and ITS 5 (5′-GGAAGTAAAAGTCGTAACAAGG-3′). The second was the calmodulin (CaM) gene, using primers Cal-228F (5-GAGTTCAAGGAGGCCTTCTCCC-3) and Cal-737R (5-CATCTTTCTGGCCATCATGG-3) [34]. The third was the elongation factor (EF) gene with primers EF-728M (5-CATYGAGAAGTTCGAGAAGG-3) and EF2 (5-GGARGTACCAGTSATCATGTT-3), following established protocols [34,35,36]. Amplicons (100 ng) from each sample were sequenced by the Sanger method at Myleus Biotechnology Company. Sequence data were trimmed using Geneious^®^ 9.0.5, and consensus sequences were analyzed for similarity using the Basic Local Alignment Search Tool (BLAST) against public databases. Taxonomic identifications at species level were only indicated after maximum likelihood phylogenetic analyses of the three genomic regions were performed (ITS, CaM, and EF) (Supplementary Materials). As *Trichoderma harzianum* is a species complex, we used *T. harzianum* (species complex), and for some isolates, *T.* cf. *asperellum*, as the identifications were based on a medium support. The alignments of each genomic region were performed using MAFFT software, version 7, checked for ambiguity among the nucleotides, and gaps were considered as missing data. We performed maximum likelihood phylogenetic analysis with IQ-TREE multicore v.1.6.12 (Supplementary Material).

### 2.2. Spore Suspension of Trichoderma spp. and A. welwitschiae

The *Trichoderma* isolates, except isolate R39, and the *A. welwitschiae* isolate were grown on potato dextrose agar (PDA) for 7 or 8 days at 28 ± 1 °C. Spore suspensions were prepared by flooding the fungal colony with 20 mL of sterilized distilled water containing two drops of Tween 20, followed by scraping with a sterile Drigalski spatula. The suspension was filtered through sterile gauze, and spores were counted using a Neubauer chamber at 400× magnification. Spore concentration was adjusted to the desired level by adding sterile distilled water.

### 2.3. Dual Culture Assays with A. welwitschiae and Trichoderma spp.

In dual culture assays, each PDA Petri dish received 5 µL of *Trichoderma* spp. spore suspension (1 × 10^5^ spores ml-¹) and 5 µL of *A. welwitschiae* spore suspension (1 × 10^5^ spores ml-¹), inoculated on opposite sides of the dish at 1 cm from the edge. Control plates contained *A. welwitschiae* inoculated on one side only. The cultures were incubated at 28 ± 2 °C in darkness, and colony diameter was measured when the control colony reached the edge of the Petri dish. Percentage inhibition (PI) was calculated as PI = [(CDAWC—CDAWP)/CDAWC] × 100, where CDAWC = colony diameter of *A. welwitschiae* in the control and CDAWP = colony diameter paired with *Trichoderma* [37]. The experimental design was completely randomized, with 18 treatments and five replicates.

For observations of biocontrol mechanisms, culture pairings were also examined for halo formation, mycelium growth, and *Trichoderma* sporulation around and over *A. welwitschiae’s* colony. To observe mycoparasitism, two sterile coverslips were placed on the PDA medium between colonies. After colony growth and contact, the coverslips were removed, placed on a microscope slide with a drop of lactoglycerol or lactoglycerol cotton blue, and they were examined at 100× magnification on a Leica DM750 microscope. The experiment was completely randomized with three replicates.

### 2.4. Volatile Compounds of Trichoderma spp. on Growth and Sporulation of A. welwitschiae

The effect of volatile compounds produced by *Trichoderma* spp. on *A. welwitschiae* was evaluated as described by [38]. Petri dishes with PDA were inoculated with 5 µL of *Trichoderma* spp. spore suspension (1 × 10^5^ spores ml-¹) and paired with an inverted PDA dish inoculated with *A. welwitschiae*. The two plates were sealed together with parafilm and incubated in the dark at 28 °C for ten days. The experimental design was completely randomized, with 18 treatments (17 isolates of *Trichoderma* and the control with *A. welwitschiae* only) and three replicates. Colony diameter and sporulation were measured as described above. Sporulation data were normalized by colony size, expressed as number of spores per cm² of colony.

### 2.5. Non-Volatile Metabolites of Trichoderma and Their Effect on A. welwitschiae

The effect of non-volatile metabolites of *Trichoderma* spp. on growth and sporulation of *A. welwitschiae* was tested with the methodology previously described [38], with a few modifications. Filtrates of the *Trichoderma* cultures were prepared by transferring five agar plugs (5 mm) of a *Trichoderma* colony to Erlenmeyer flasks containing 250 mL of potato dextrose broth, and they were incubated at 28 °C with orbital agitation (120 rpm) for 7 days. After incubation, the cultures were filtered through filter paper (0.20 mm thick, which retains particles around 4–7 µm) and centrifuged at 2550 rpm for three minutes for the removal of fungal mycelium and spores. Fifteen milliliters of the culture filtrate were added to 45 mL of sterile PDA medium at the melting point, and the medium was homogenized through manual agitation and transferred to Petri dishes. The control treatment consisted of PDA without the culture filtrate. The center of the plates was inoculated with 5 µL of *A. welwitschiae* spore suspension (1 × 10^5^ spores ml^−1^). All cultures were incubated in the dark at 28 °C for 8 days. Measurements of colony diameter and sporulation were performed as already described for volatile compounds. The experimental design was completely randomized, with 18 treatments and four replicates.

For evaluation of the effect of non-volatile metabolites on *A. welwitschiae* spore germination, 96-well ELISA type microplates were used with 100 μL of *A. welwitschiae* spore suspension (1 × 10^5^ spores ml^−1^), 100 μL of *Trichoderma* spp. filtrate and 50 μL of potato dextrose broth in each plate well [39]. The control treatment consisted of 100 μL of the spore suspension of *A. welwitschiae* and 150 μL of potato dextrose broth. All plates were incubated in the dark at 28 °C for 24 h. The control treatment was evaluated for spore germination, and when they reached 70% germination, 5 μL of lactoglycerol blue was added to all wells to stop spore germination. For each well, the spores were transferred with a micropipette to a microscope slide, and 100 spores were counted, considering germinated and non-germinated spores, under a microscope with 400× magnification. Spores were considered germinated when their germinating tube reached a length that was at least two times the spore size. The experimental design was completely randomized, with 18 treatments and seven replicates (each well was a replicate of the treatment).

### 2.6. Control Assays of Bole Rot Disease Under Greenhouse Conditions

#### 2.6.1. Growth of Sisal Plants

Sisal bulbils collected in the field in the municipality of Conceição do Coité (12°39′41″ S and 39°5′12″ W), Bahia, Brazil, were planted in plastic trays with soil and were irrigated every 3 or 4 days. After 60 days, the sisal bulbils were transplanted into perforated black plastic bags measuring 18 × 24 cm, each filled with 2 L of soil. They were then cultivated in a controlled greenhouse environment until they attained a height ranging from 28 to 30 cm. Subsequently, these plants were inoculated for bole rot control experiments as follows.

#### 2.6.2. Trichoderma and *A. welwitschiae* Inoculation Assay in Sisal Plants

To explore the potential of *Trichoderma* for controlling bole rot disease caused by *A. welwitschiae* in sisal plants, 17 isolates detailed in Table 1 were tested. Isolate R39 was not used because of the limited number of plants available for this experiment. Sisal plants underwent incisions, creating two micro-holes on opposite sides of the stem, using a sterile needle from a disposable sterile syringe (25 × 0,70 mm). Each micro-wounded plant stem was inoculated with 1 mL of a spore suspension (1 × 10^7^ spores ml^-1^) of *Trichoderma* sp., allowing any excess inoculum to flow through the outer stem tissue. After 24 h, the micro-wounded stem regions were further inoculated with 1 mL of a spore suspension (1 × 10^7^ spores ml^-1^) of *A. welwitschiae*. The following control treatments were included: (i) sisal plants solely inoculated with *A. welwitschiae* (without *Trichoderma*); (ii) sisal plants exclusively inoculated with *Trichoderma* isolate TCS15 (without *A. welwitschiae*); (iii) sisal plants exclusively inoculated with *Trichoderma* isolate TCS35 (without *A. welwitschiae*) and (iv) sisal plants that received 1 mL of distilled water in the micro-wounded stem (without inoculation). The experimental design was in random blocks with three replicates and ten plants per replicate.

Disease incidence and severity were evaluated 30 days after inoculation with *A. welwitschiae* by removing the plants from the plastic bags and cutting them longitudinal for evaluation of external and internal symptoms of sisal bole rot. The disease scale developed by Barbosa [39] was used. The percentage of disease incidence was calculated based on the number of plants with bole rot symptoms, divided by the total number of plants for each treatment. Data of disease severity were used to calculate disease index, with the equation: Σ (DSN * F) * 100/(NE * MaxDSN), with DSB = disease scale note; F = frequency; NE= number of evaluations; MaxDSN = maximum disease scale note [40].

The efficiency of disease control was calculated with the equation: E = [(DICP − DIPT)/DICP] × 100, with E = Efficiency; DICP = disease incidence of control plants inoculated with the pathogen; DIPT = disease incidence of the plants inoculated with the isolates of *Trichoderma* and the pathogen.

#### 2.6.3. Anatomical Analysis of Sisal Bole Tissue

Sisal stem tissue was collected for anatomical analysis at the end of the experiment (30 days after pathogen inoculation), except in the case of plants exclusively inoculated with *A. welwitschiae*. For those plants, the stem tissue was cut 15 days after pathogen inoculation because the plant already showed severe rot tissue and death.

Stem tissue fragments were immersed in FAA 70 solution (Formaldehyde 37%; ethanol 70%; glacial acetic acid, 0.5:0.5:9 volume/*v*/*v*) for 24 h [41]. A vacuum was applied during the fixation process, and subsequently, the samples were stored in 70% ethanol for preservation. The tissue samples were dehydrated in an ethanol series and were inserted in 2-hidroxyethylmetha-acrilatum (historresin-Leica), as described previously [42]. Transversal and longitudinal sections of stem tissue with thickness varying from 10 to 12 µm were obtained with a rotative microtome (Leica RM2245). The tissue cuts were double stained with toluidine and basic fuchsin [43]. The microscope slides were mounted with synthetic resin (Permount/Fisher) and then analyzed under an Olympus BX51 microscope equipped with a digital photographic camera (Olympus A330).

### 2.7. Data Analysis

The data were analyzed for normality with the test of Shapiro–Wilk. Data of sporulation (volatile and non-volatile compounds) were transformed in log. Untransformed data values were used for the graphs presented in this paper. Analysis of variance by the F test was performed, and for treatments with significant differences, means were compared by the test of Scott–Knott (*p* ≤ 0.05). The statistical software R version 4.4.2 [44] was used. In addition, multivariate statistical analyses were performed at MetaboAnalyst 5.0 “(http://www.metaboanalyst.ca, accessed on 27 September 2023)” by using the default settings [45] and encompassed principal component analysis (PCA), hierarchical cluster analysis, and heat map. For the hierarchical cluster analysis, we used the Euclidean coefficients as distance measure and Ward as the clustering algorithm. The circles around samples in the PCA plot portray the 95% confidence region of the replicates.

## 3. Results

### 3.1. Molecular Identification of Trichoderma Isolates and A. welwitschiae

Three nuclear regions were used to identify *Trichoderma* isolates at the species level. *T. harzianum* (species complex), *T*. cf. *asperellum*, and five other species were successfully identified, with *T. harzianum* (species complex), *T. lentiforme*, and *T.* cf. *asperellum* emerging as the most prevalent among them. Among these, *T. harzianum* (species complex) and *T. lentiforme* were the predominant ones isolated from the soil and roots of sisal plants from the semi-arid region of Bahia, Brazil. Information about the taxonomic identification of all *Trichoderma* isolates, the GeneBank accession numbers, as well as their habitat are listed in Table 1. *A. welwitschiae* was identified in a previous work [14], and the accession number is also in Table 1.

### 3.2. Dual Culture Assays with Trichoderma and A. welwitschiae

**Figure 1:**
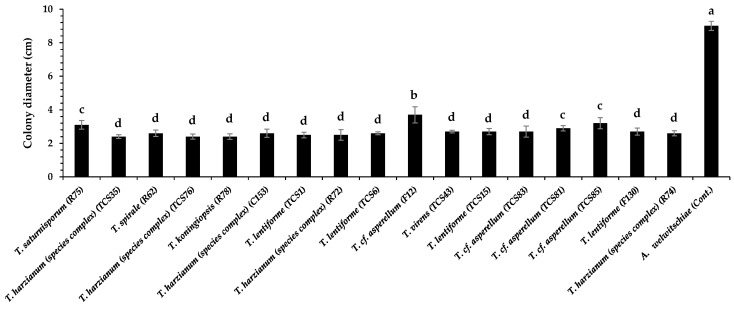
Colony diameter of *Aspergillus welwitschiae* paired with *Trichoderma* spp. in PDA medium. Bars with same letter do not differ statistically by the test of Scott–Knott (*p* ≤ 0.05).

All *Trichoderma* isolates significantly inhibited the mycelium growth of *A. welwitschiae* (*p* ≤ 0.05; Figure 1). Inhibition rates against *A. welwitschiae* varied from 58 to 73%, with *T.* cf. *asperellum* (F12) being the less efficient isolate, with 58% growth inhibition (Figure 1).

**Figure 2:**
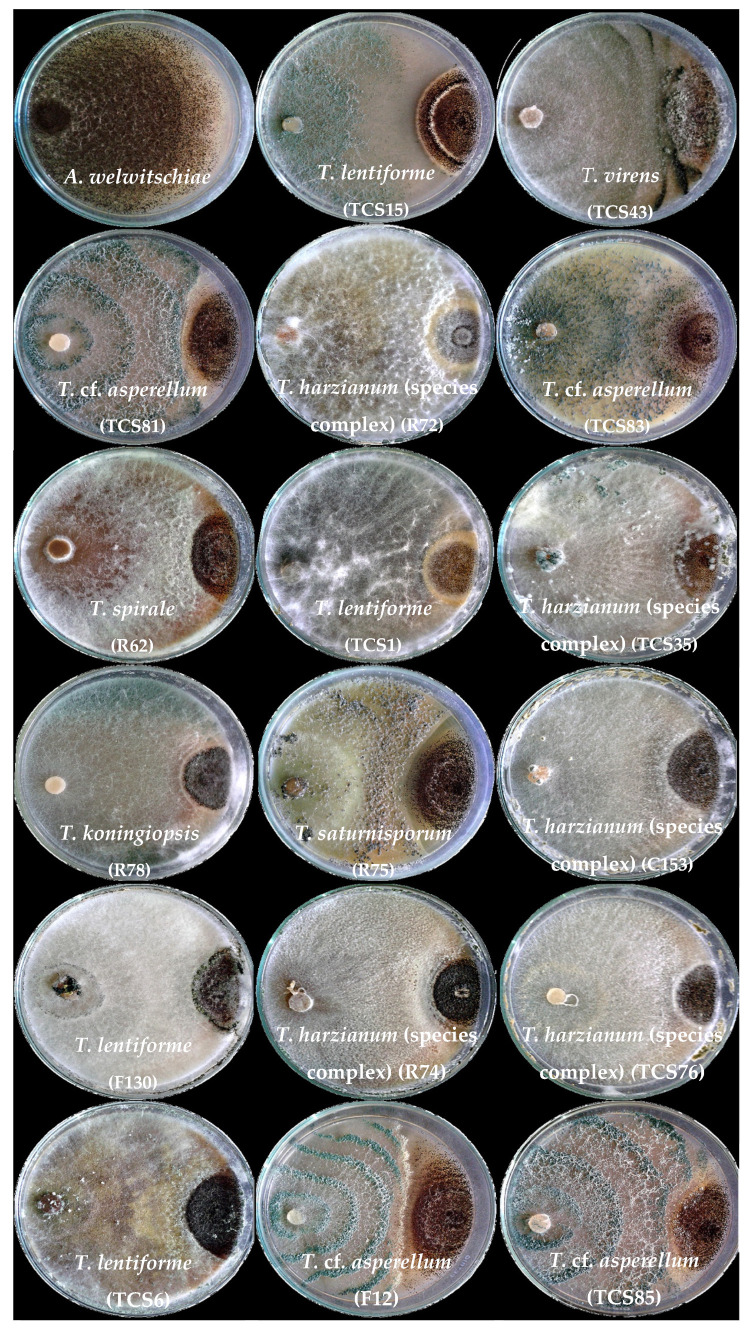
Interaction between isolates of *Trichoderma* and *A. welwitschiae* in paired cultures. Except for the control with *A. welwitschiae* only, all paired cultures have *Trichoderma* on the left side and *A. welwitschiae* on the right side.

**Figure 3:**
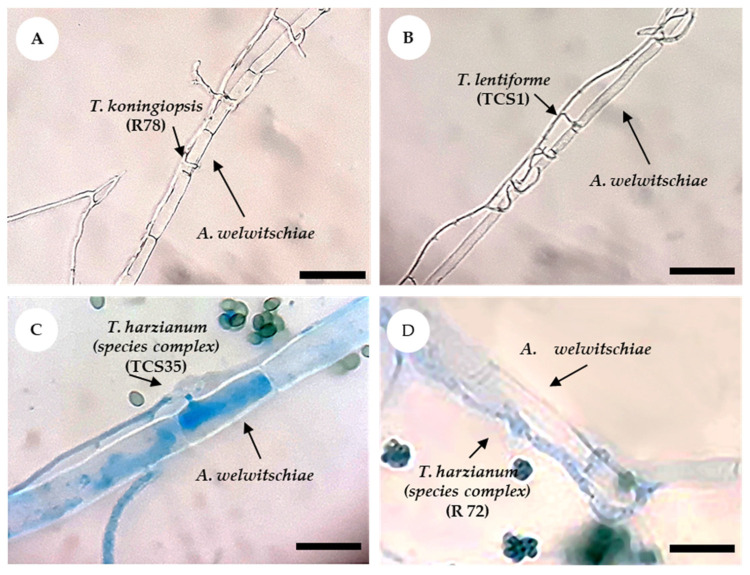
Interaction between isolates of *Trichoderma* and *A. welwitschiae*, observed under a microscope with 1000×. (**A**,**B**) Unstained slides showing *Trichoderma* growth and coiling around the hyphae of *A. welwitschiae*, with hyphae strangulation. (**C**,**D**) Stained slides showing *Trichoderma* on *A. welwitschiae*’*s* hyphae, with the formation of appressorium-like structures and penetration into *A. welwitschiae’s* hyphae. Scale: 50 µm (**A**,**B**); 20 µm (**C**,**D**).

Most isolates exhibited widespread growth across the plate and overlapped the *A. welwitschiae* colony (Figure 2). Notably, only the sisal-associated isolates *T. lentiforme* (TCS1), *T. harzianum* (species complex) (TCS35 and R72), and *T. koningiopsis* (R78) exhibited mycoparasitism behavior against *A. welwitschiae* (Figure 3). The hyphae of *T. koningiopsis* (R78) and *T. lentiforme* (TCS1) grew around *A. welwitschiae*’s hyphae, with hyphae coiling and strangulation (Figure 3A,B). The formation of appressorium-like structures and penetration into *A. welwitschiae*’s hyphae was observed with *T. harzianum* (species complex) (TCS35 and R72) (Figure 3C,D).

### 3.3. Effect of Volatile Compounds of Different Species and Isolates of Trichoderma spp. on Growth and Sporulation of A. welwitschiae

**Figure 4:**
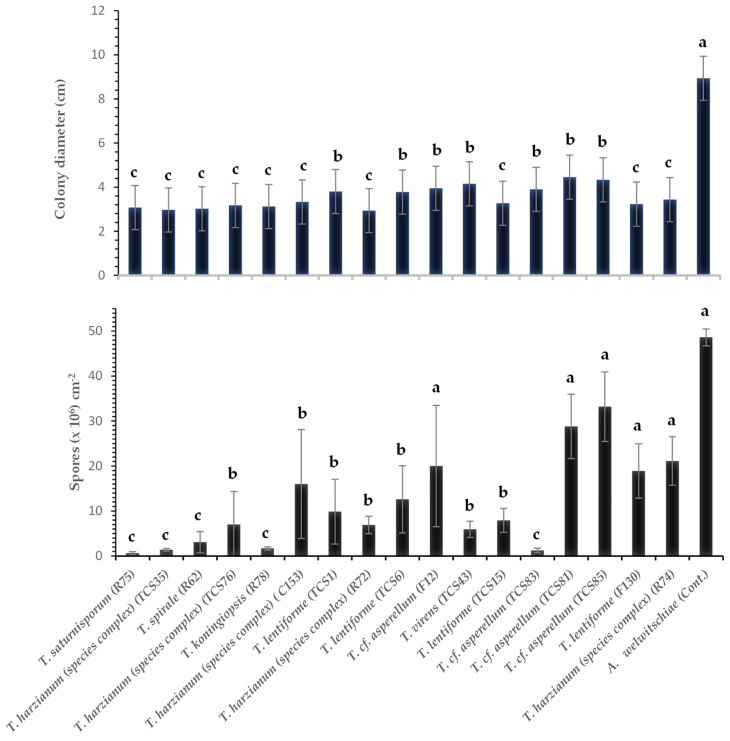
Colony diameter (cm) and sporulation (number of spores per cm^2^ of colony) of *A. welwitschiae* affected by volatile compounds produced by *Trichoderma* spp. Bars with the same letters do not differ statistically by the test of Scott–Knott (*p* ≤ 0.05).

**Figure 5:**
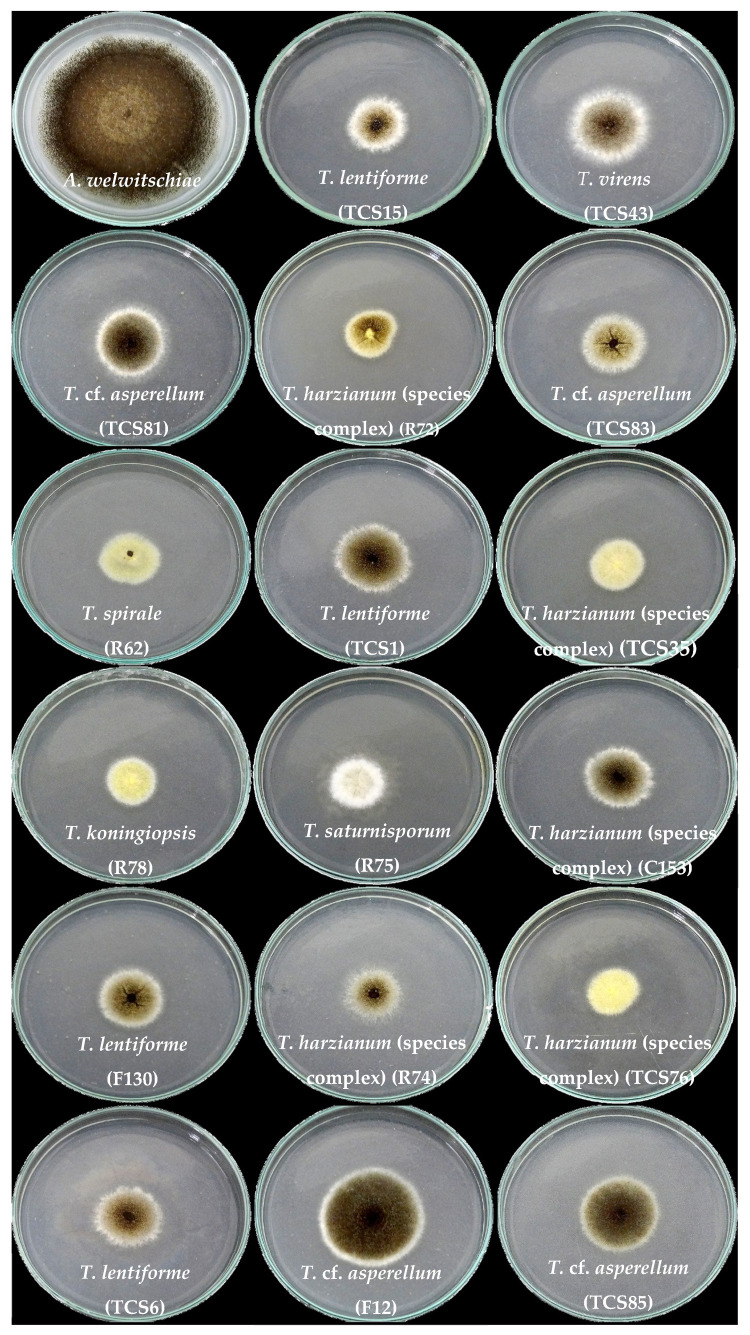
*A. welwitschiae* exposed to volatile compounds produced by *Trichoderma* spp. The culture with *A. welwitchiae* without *Trichoderma* spp. refers to the control treatment.

Volatile compounds of Trichoderma inhibited between 50 and 66% of A. welwitschiae’s colony growth. All treatments were significantly different (*p* ≤ 0.05) from the control (without Trichoderma), which showed an average colony diameter of 8.93 cm (Figure 4 and Figure 5).

Additionally, the sporulation of *A. welwitschiae* was influenced by volatile compounds produced by most of the *Trichoderma* isolates. Volatile compounds secreted by *T.* cf. *asperellum* (TCS81, TCS85, and F12), *T. harzianum* (species complex) (R74), and *T. lentiforme* (F130) did not significantly inhibit sporulation of *A. welwitschiae*. On the other hand, volatile compounds from *T. harzianum* (species complex) (TCS35), *T.* cf. *asperellum* (TCS83), *T. spirale* (R62), *T. saturnisporum* (R75), and *T. koningiopsis* (R78) led to the most significant reduction in *A. welwitschiae* sporulation (Figure 4 and Figure 5).

### 3.4. Nonvolatile Compounds (Culture Filtrates) of Trichoderma spp. Against A. welwitschiae

**Figure 6:**
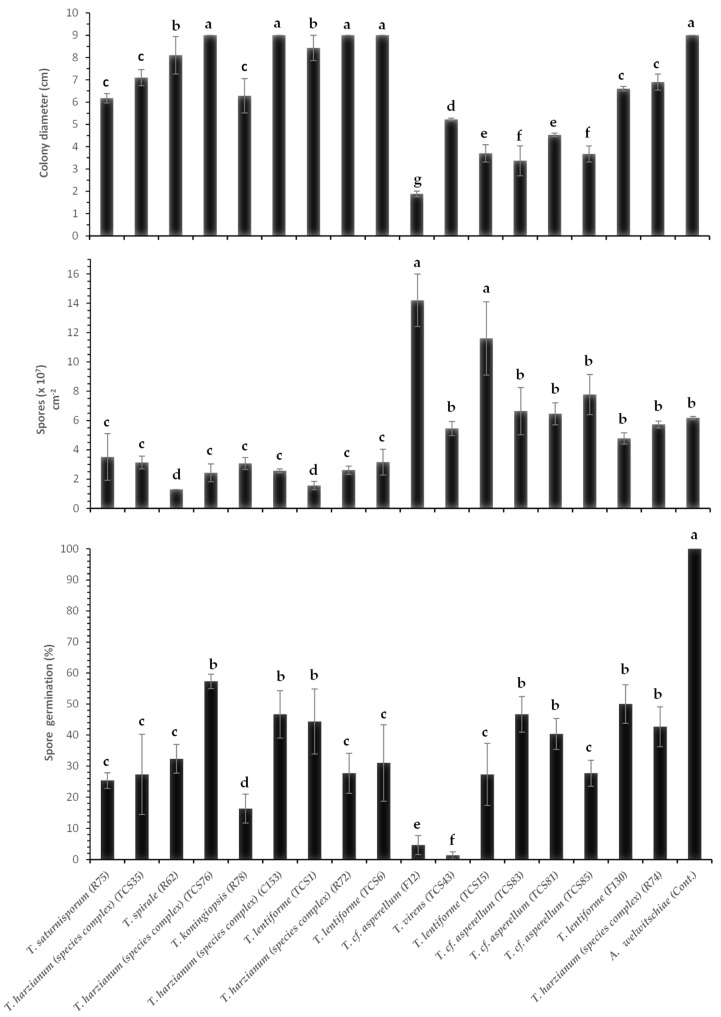
Inhibitory effect of non-volatile compounds produced by *Trichoderma* spp. on colony diameter (cm), sporulation (number of spores per cm^2^ of colony), and spore germination (%) of *A. welwitschiae.* Bars with the same letters do not differ statistically by the test of Scott–Knott (*p* ≤ 0.05).

The impact of non-volatile compounds on the mycelium growth of A. welwitschiae differed among the Trichoderma isolates. T. harzianum (species complex) (C153, R72, and TCS76) and T. lentiforme (TCS1) did not affect mycelium growth through production of non-volatile compounds. In contrast, T. cf. asperellum (F12) caused significant inhibition (80%) of A. welwitschiae growth (Figure 6).

The sporulation of A. welwitschiae was also affected by non-volatile compounds. The lowest sporulation of A. welwitschiae was obtained for the treatments with T. lentiforme (TCS1) and T. spirale (R62) with 1.58 × 10^7^ and 1.31 × 10^7^ spores cm^−2^ of fungal colony, respectively (Figure 6). On the other hand, T. lentiforme (TCS15) and T. cf. asperellum (F12) stimulated the pathogen’s sporulation, with 9.86 × 10^7^ and 1.46 × 10^8^ spores cm^−2^ of fungal colony, as compared to the control (6.02 × 10^7^ spores cm^-2^). In addition, the non-volatile compounds inhibited spore germination (Figure 6). Spores of A. welwitschiae, without the effect of Trichoderma, showed 100% germination. In contrast, T. virens (TCS43) and T. cf. asperellum (F12) caused a reduction of 99% and 97% of the pathogen’s spore germination, respectively (Figure 6).

### 3.5. Bole Rot Control in Sisal Bulbils Under Greenhouse Conditions

**Figure 7:**
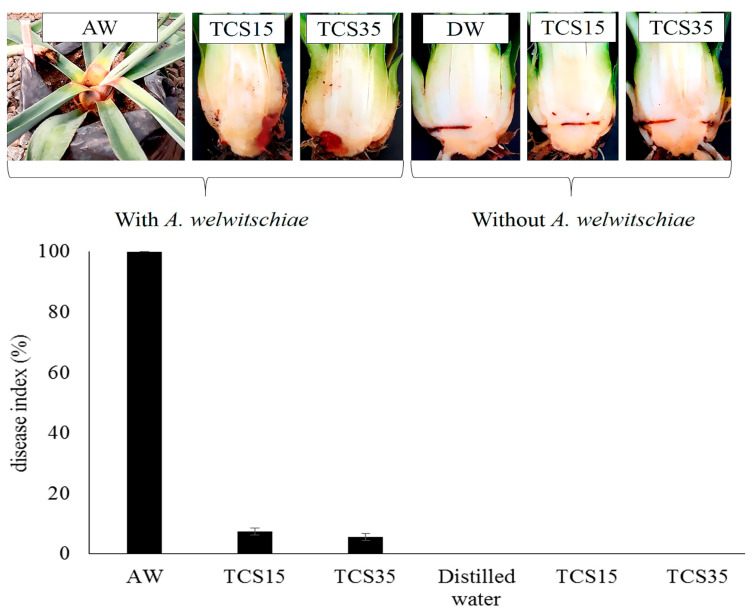
Disease index of sisal bole rot in plants treated with *T. harzianum* (species complex) (TCS35) and *T. lentiforme* (TCS15) and inoculated with *A. welwitschiae* (AW). These were compared to plants without *Trichoderma* but inoculated with *A. welwitschiae* (AW), plants with wounded bole tissue but treated only with *Trichoderma*, and those that received solely distilled water (DW).

**Figure 8:**
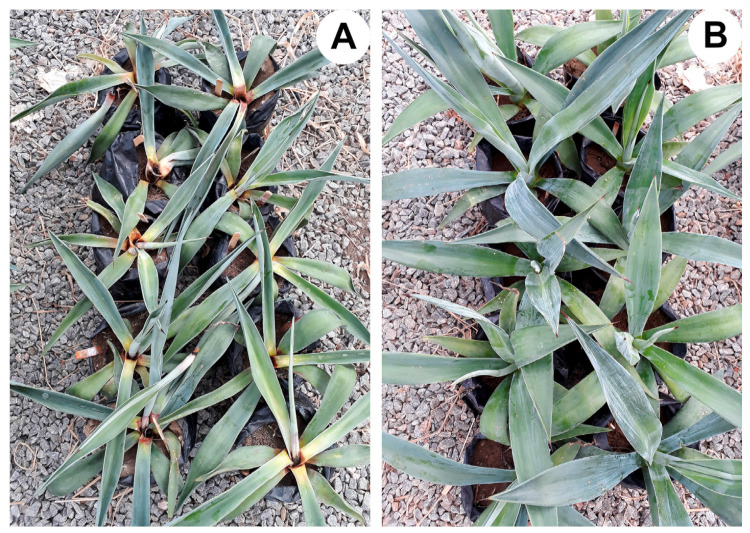
Sisal plants at 30 days after inoculation with *A. welwitschiae*. (**A**) Plants inoculated only with *A. welwitschiae* (control treatment) showing complete bole and leaf base rot, with plant death. (**B**) Plants treated with *T. harzianum* (species complex) (TCS35) and inoculated with *A. welwitschiae*.

**Figure 9:**
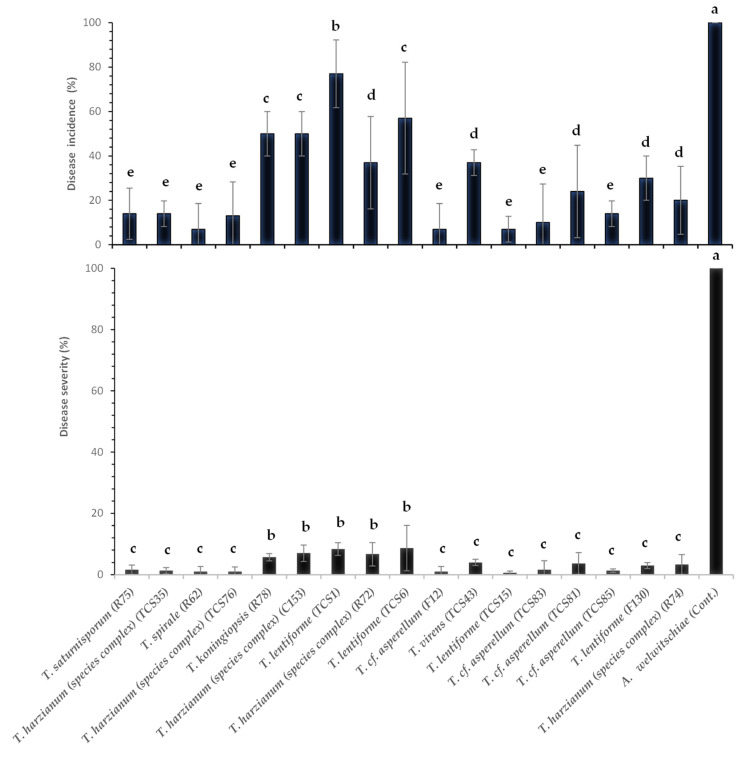
Sisal bole rot incidence (%) and disease index (%) in sisal plants treated with *Trichoderma* spp. and inoculated with the pathogen *A. welwitschiae*. Means followed by the same letter in the same column are not statistically different, by Scott–Knott (*p* ≤ 0.05).

All *Trichoderma*-treated sisal plants exhibited a significant reduction (*p* < 0.05) in disease incidence and severity over a 30-day evaluation period following pathogen inoculation (Figure 7, Figure 8 and Figure 9). Figure 7 illustrates the outcomes of a preliminary experiment conducted with sisal plants involving only two *Trichoderma* isolates before the comprehensive experiment with all isolates. Control treatment plants (inoculated with *A. welwitschiae* but without *Trichoderma*) displayed 100% disease incidence, characterized by yellowing and wilting leaves, with bole and leaf base rot (Figure 8A). These symptoms were initiated on the 8th day after inoculation with *A. welwitschiae*, and within 15 days, approximately 90% of the plants were dead with completely rotted boles and leaf basal parts (Figure 8A). However, plants treated with *Trichoderma* exhibited disease symptoms considerably later, within 20 days after pathogen inoculation. Furthermore, for the majority of *Trichoderma* treatments, the plants remained free from bole rot symptoms throughout the entire period of the experiment (Figure 8B). It was also observed that sisal plants that were wounded in the bole tissue but were not inoculated with *A. welwitschiae* showed red tissue only localized around the wounds, that did not evolve to bole rot symptoms, which indicates that disease symptoms and plant death were caused by *A. welwitschiae* infection (Figure 7).

Plants treated with *T. lentiforme* (TCS1) demonstrated the lowest efficiency in bole rot control, resulting in a 77% disease incidence. In contrast, *Trichoderma lentiforme* (TCS15 and T130), *T. harzianum* (species complex) (TCS76, TCS35, and R74), *T.* cf. *asperellum* (TCS81, TCS83, TCS85, and F12), *T. spirale* (R62), and *T. saturnisporum* (R75) effectively reduced disease incidence by 70% to 93% and disease severity by 97% (Figure 9). This remarkable disease control was consistently observed in plants treated with the aforementioned *Trichoderma* isolates (Figure 8B).

**Figure 10:**
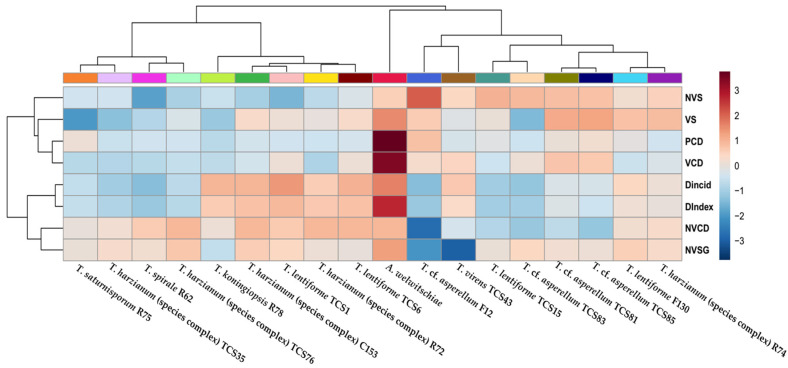
Heat map with a dendrogram of in vitro tests for control of sisal bole rot with *Trichoderma* spp., as well as disease incidence and severity index of sisal plants treated with *Trichoderma* spp. and *Aspergillus welwitschiae*. The dendrogram on the left side depicts relationships between disease incidence and severity index, whereas the dendrogram on top depicts the relationships between *Trichoderma* spp. and *A. welwitschiae*. Dark red indicates higher values while dark blue indicates lower values. NVS—non-volatile compounds and sporulation. VS—volatile compounds and sporulation; PCD—paired colony diameter; VCD—volatile compounds and colony diameter; DIncid—disease incidence; DIndex—disease index; NVCD—non-volatile compounds and colony diameter; NVSG—non-volatile compounds and spore germination.

To better understand the relationship between the isolates of *Trichoderma*, their mechanisms of action, and disease control, we generated a heat map and a dendrogram for the variables analyzed (Figure 10). The heat map accompanied by the dendrogram reveals a distinct separation of *Trichoderma* isolates into two clusters. One cluster is composed of *T.* cf. *asperellum* (F12, TCS81, TCS83, and TCS85), *T. virens* (TCS43), *T. lentiforme* (TCS15 and F130), and *T. harzianum* (species complex) (R74), as well as *A. welwitcheae*. Notably, isolates F12, TCS15, and TSC83 can be pointed out as the most effective ones for sisal bole rot control (Figure 10). Mycelium inhibition by non-volatile compounds appears to be an important mechanism of action of these isolates. For *T. virens* (TCS43), although it showed the strongest inhibition of spore germination by non-volatile compounds, it had a weaker effect on inhibition of mycelium growth and was less effective in controlling disease incidence and severity. Although *T. lentiforme* (TCS15) and *T.* cf. *asperellum* (TCS83) exhibited a milder impact on inhibition of spore germination, these isolates demonstrated a more pronounced effect in suppressing *A. welwitcheae* mycelium growth, as well as disease incidence and severity. *T.* cf. *asperellum* (TSC81 and TSC85) displayed limited effectiveness in inhibiting spore germination and achieved mycelium growth inhibition through non-volatile compounds. However, their performance in disease control was comparatively less efficient.

The other cluster is composed of *T. lentiforme* (TCS6 and TCS1), *T. harzianum* (complex species) (R72, C153, TCS76, and TCS35), *T. koningiopsis* (R78), *T. spirale* (R62), and *T. saturnisporum* (R75). Within this cluster, *T. spirale* (R62), *T. harzianum* (species complex) (TCS35 and TCS76), and *T. saturnisporum* (R75) were the most efficient ones in controlling bole rot disease. Mycelium inhibition by volatile compounds and through culture pairing, as well as inhibition of sporulation by both volatile and non-volatile compounds, seem to be the important mechanisms of these isolates. The other isolates, although having some in vitro mechanisms of fungal control, were not efficient in controlling bole rot disease, as compared to the ones listed above.

**Figure 11:**
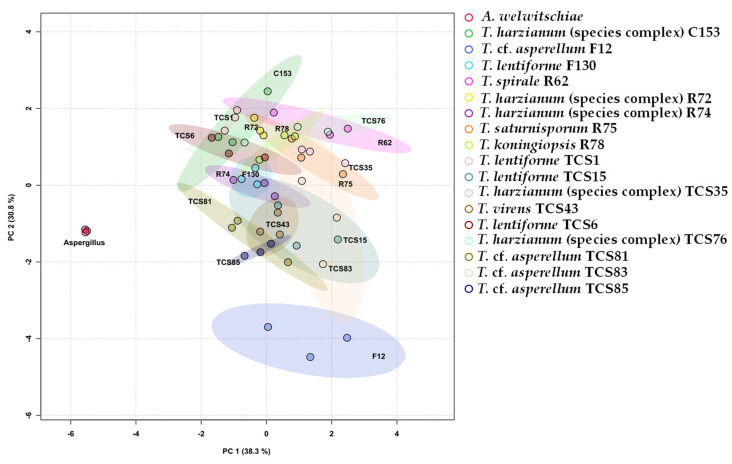
Scores plot and two component analysis of the in vitro tests for biocontrol of sisal bole rot with *Trichoderma*, as well as disease incidence and severity index of sisal plants treated with different isolates of *Trichoderma* and with *Aspergillus wewitschiae*.

Another analysis for a better understanding of the data is the principal components analysis (PCA) and scores plot, which revealed a clear distinction between the control treatment (plants inoculated with *A. welwitcheae* without *Trichoderma*), and other clusters related to different isolates of *Trichoderma* and their action in controlling *A. welwitschiae* (Figure 11). According to the PCA, 69.1% of the results can be explained by the variables analyzed. The outstanding effect of *T.* cf. *asperellum* (F12) is noticeable, with a unique cluster, and the strongest effect on inhibition of sporulation by non-volatile compounds can be observed in the heat map. It also becomes clear the two larger clusters shown in the heat map with a dendrogram (Figure 10), with *T. lentiforme* (TCS15), *T.* cf. *asperellum* (TCS83, TCS85, and TCS81) being differentiated by their mechanisms of action, while *T. harzianum* (species complex) (TCS35 and TCS76), *T. saturnisporum* (R75), and *T. spirale* (R62) were differentiated by other mechanisms. Furthermore, *T.* cf. *asperellum* (F12) can be pointed out as the most consistent and efficient one in controlling sisal bole rot.

### 3.6. Anatomical Analysis of Sisal Bole Rot Tissue

**Figure 12:**
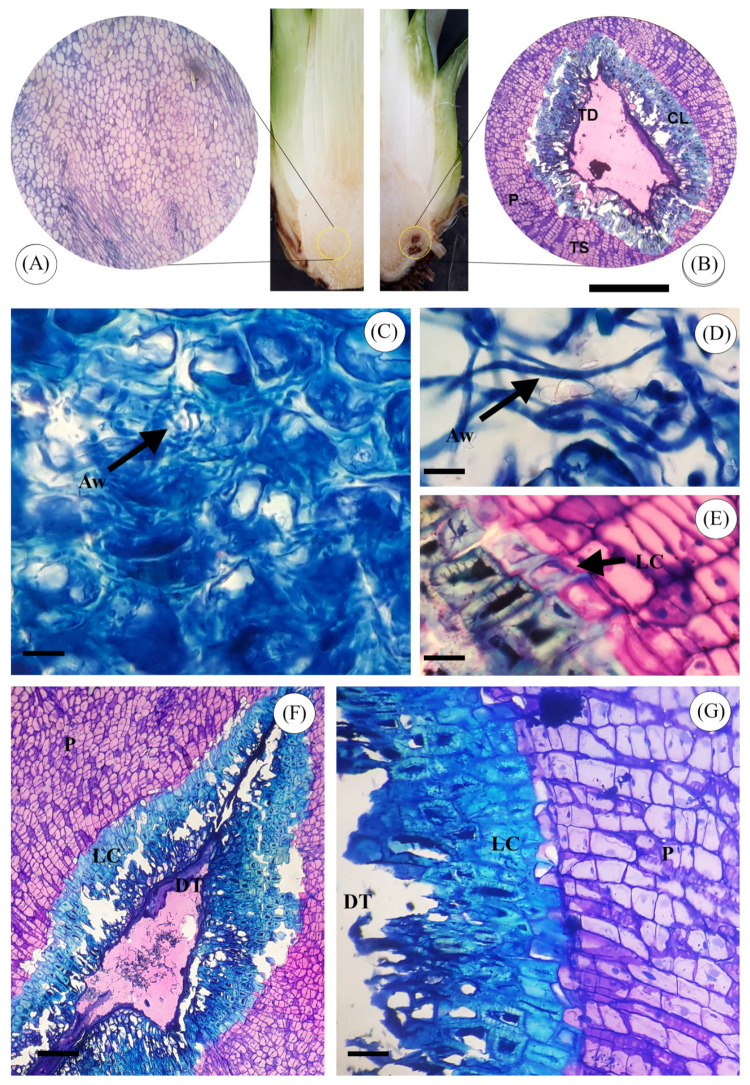
Transversal sections of sisal bole inoculated with *Trichoderma* spp. and *A. welwitschiae*. (**A**) Healthy sisal bole tissue (noinoculation). (**B**) Sisalbole tissue inoculated with *Trichoderma* and *A. welwitschiae*. (**C**,**D**) Note the presence of massive *A. welwitschiae* hyphae (arrow) in infected and rot bole tissue. (**E**) Parenchyma cells with increased thickness of cell walls (arrow). Tissue double-stained with toluidine blue and basic fuchsin; the blue to green color indicates an affinity for acid structures such as lignin deposited in the cell walls. (**F**,**G**) Lignification of parenchyma cellsaround damaged bole tissue folowing infection by *A. welwitschiae*. Samples stained with toluidine blue; cell walls rich in pectin exhibit purple color, cellulosic cell walls stain blue, and lignified cell walls can exhibit blue to green colors. Abbreviations: Aw—*Aspergillus welwitschiae*; LC—Lignified Cells; P—Parenchyma; DT—Dead Tissue. Disease Scale: 150 µm (**F**); 60 µm (**A**,**B**); 1 µm (**C**–**E**,**G**).

The anatomical characteristics of sisal bole tissue are illustrated in Figure 12. In the absence of any inoculation (Figure 12A), bole healthy tissue was composed of iso-diametric parenchyma cells with thin cell walls. No pre-existing plant defense mechanisms were observed. In contrast, bole tissue inoculated solely with *A. welwitschiae*, without the presence of *Trichoderma* spp., displayed deteriorated cells, lacking a defined shape. Furthermore, it showed intense colonization of *A. welwitschiae* throughout the tissue (Figure 12C,D). In the anatomical sections of boles treated with *Trichoderma* spp. and subsequently inoculated with the pathogen, a defense mechanism was observed, with the formation of a physical barrier, resulting in an increased thickness of the cell wall around infected tissue. This structural modification effectively prevented further tissue colonization by *A. welwitschiae* (Figure 12B,E–G).

## 4. Discussion

Sisal bole rot disease is the leading phytosanitary challenge across all sisal-producing regions in Brazil [10,15]. Given the lack of established control methods and the pathogen’s considerable destructiveness, developing effective control strategies is urgently needed. In this study, we evaluated the potential of *Trichoderma* spp. as biocontrol agents against *A. welwitschiae* and examined whether the geographical origin and lifestyle (soil inhabitant or endophyte) of *Trichoderma* isolates influenced their efficacy.

Our findings demonstrate that *Trichoderma* can effectively control sisal bole rot through mechanisms such as competition, mycoparasitism, and the production of antifungal volatile and non-volatile compounds, which inhibited mycelial growth, sporulation, and/or spore germination of *A. welwitschiae*. The isolates were divided into two clusters based on their biocontrol approach. While tests using dual culture and antibiosis through secondary metabolites might overestimate some mechanisms and underestimate others [46], our study indicates that these tests can effectively categorize *Trichoderma* isolates based on their biocontrol strategies (Figure 10). Notably, inhibition of mycelial growth and spore germination by non-volatile compounds were significant mechanisms for the most effective isolates, namely *T.* cf. *asperellum* (F12 and TCS83) and *T. lentiforme* (TCS15). Similarly, *T. harzianum* (species complex) (TCS35 and TCS76), *T. spirale* (R62), and *T. saturnisporum* (R75) were effective in controlling sisal bole rot through mechanisms involving volatile compounds and culture pairing.

The volatile compounds produced by *T. harzianum* have been shown to inhibit the growth of *Aspergillus flavus* [47]. However, this capability appears to vary with *Trichoderma* species and strain, which is consistent with our observations of *T. harzianum* (species complex) and *T.* cf. *asperellum*. Additionally, *T. spirale* has demonstrated control over pathogens like *Fusarium oxysporum* and has successfully been used for *Fusarium* wilt management of cherry tomato plants in greenhouse settings [48]. *T. saturnisporum*, known as a growth-promoting fungus for nursery plants [49], also produces β-endoglucanase, which inhibits *F. oxysporum* [50]. In our study, the inhibition of *A. welwitschiae* by different isolates of *T.* cf. *asperellum* involved non-volatile compounds. Secondary metabolite production by *Trichoderma* is known to depend on the isolate and environmental conditions, which may not mirror in-situ production [46].

Significantly, the most effective *Trichoderma* isolates against *A. welwitschiae* originated from diverse ecological niches (Table 1, Figure 10 and Figure 11). These isolates included endophytes from sisal roots (*T. saturnisporum* R75 and *T. spirale* R62) and leaves (*T.* cf. *asperellum* F12) and isolates from soil surrounding sisal roots (*T. lentiforme* TCS15 and *T. harzianum* (species complex) TCS76 and TCS35), in the Caatinga biome of Bahia, Brazil. Promising isolates were also obtained from the soil surrounding banana roots in an irrigated banana field in Bahia’s semi-arid region (*T.* cf. *asperellum* TCS83) and from leaves of a guava plant located in our university campus of Cruz das Almas city, which is in Bahia’s Atlantic Forest biome (*T.* cf. *asperellum* TCS81). This ecological diversity reflects the range of mechanisms utilized by these isolates to control *A. welwitschiae*. Despite the different origins (soil or endophyte), sisal plants inoculated through wounded bole tissue thrived, indicating that these *Trichoderma* isolates can adapt to various environments. Indeed, *T.* cf. *asperellum* (F12) has been shown to survive in multiple soil samples from sisal-producing areas (unpublished data). *Trichoderma*-plant interactions typically occur in the root zone, encompassing both external and internal root parts [28]. However, some species colonize various plant tissues beyond roots, as seen with *T. amazonicum* in *Hevea* leaves [51], *T. atroviride,* and *T. koningii* in *Cupressaceae* leaves [52], and others in diverse plant parts [53].

Regarding other mechanisms, *Trichoderma* overgrowth on *A. welwitschiae* colonies was observed, but only *T. lentiforme* (TCS1), *T. harzianum* (species complex) (TCS35 and R72), and *T. koningiopsis* (R78) exhibited mycoparasitism, with only *T. harzianum* (species complex) (TCS35) showing effective control of bole rot disease in sisal plants, suggesting that mycoparasitism is not the primary mechanism of *A. welwitschiae* control. While mycoparasitism of *Trichoderma* on other pathogens is well documented [18,50,54,55,56], it has not been shown before with *A. welwitschiae* or the *A. welwitschiae–A. sisalana* pathosystem.

Our anatomical analysis revealed that inoculation with *Trichoderma* induced cell wall thickening and lignification in parenchyma cells surrounding infected bole tissue (Figure 12E–G), which prevented tissue colonization by *A*. *welwitschiae*. This defense response, possibly involving lignin deposition, aligns with findings in other crops where *Trichoderma* stimulates plant defense mechanisms [57,58]. For example, lignin and callose buildup is associated with *T. hamatum* defenses against *S. graminicola* in millet [59], and similar responses have been observed in other plants.

Furthermore, sisal plants exhibited enhanced structural defenses when wounded, particularly when treated with *Trichoderma*, producing red discoloration around the wounded tissue, indicating a defense response involving pigment production. Non-treated plants could not prevent *A. welwitschiae* colonization, and bole rot advanced rapidly, causing plant death. These findings suggest that *Trichoderma* may stimulate rapid lignin deposition in the parenchyma tissue, preventing pathogen penetration and colonization. This intense defense response of *A. sisalana* when treated with *Trichoderma* may be a key mechanism for boosting disease resistance. For future studies, investigating the specific defense mechanisms in *A. sisalana* and related species could lead to a deeper understanding of *Trichoderma*-induced resistance in agaves. Differences between the tested *Trichoderma* isolates in inducing plant defense mechanisms were not observed, which should also be further investigated.

In this study, we have shown that the effective control of sisal bole rot by different species and isolates of *Trichoderma* can be a result of a combination of mechanisms of induced plant defense responses along with mechanisms that cause inhibition of *A. welwitschiae’s* growth, sporulation and/or spore germination through the production of volatile and non-volatile compounds and/or through mycoparasitism.

## 5. Conclusions

Several *Trichoderma* species, including native semi-arid strains, non-native strains, and isolates from other hosts as either soil inhabitants or endophytes, demonstrated effective control over sisal bole rot disease. The most efficient isolates were *T.* cf. *asperellum* (F12 and TCS83), *T. lentiforme* (TCS15), *T. harzianum* (species complex) (TCS35 and TCS76), *T. spirale* (R62), and *T. saturnisporum* (R75). Plant defense induction through cell wall thickening, possibly via lignification, was promoted by several *Trichoderma* isolates, warranting further investigation. Additionally, exploring alternative inoculation methods beyond bole tissue wounding will be crucial for field applications. This study marks the first report of sisal bole rot control by various *Trichoderma* species and the first anatomical investigation of the *A. sisalana-Trichoderma-A. welwitschiae* interaction. The potential use of *Trichoderma* in sisal production could substantially enhance sustainability, particularly benefiting family-based farmers who are Brazil’s primary sisal producers, as well as other fiber producers globally.

## Figures and Tables

**Table 1:** Isolates of *Trichoderma* spp.

Strain Code	Identification	GeneBank Number			
		ITS	*Tef1*	*Cal1*	Habitat
TCS1	*T. lentiforme*	MW579424	PQ062204	PQ083839	Soil from sisal field
TCS6	*T. lentiforme*	MW579426	PQ062206	PQ083841	Soil from sisal field
TCS15	*T. lentiforme*	MW579430	PQ062210	PQ083845	Soil from sisal field
TCS43	*T. virens*	MW579428	PQ062208	PQ083843	Soil from sisal field
TCS35	*T. harzianum* (species complex)	MW579427	PQ062207	PQ083842	Soil from sisal field
TCS76	*T. harzianum* (species complex)	MW579429	PQ062209	PQ083844	Soil from sisal field
TCS81	*T.* cf. *asperellum*	PQ555284	PQ569841	PQ569839	Guava plant leaf
TCS83	*T.* cf. *asperellum*	MW579425	PQ062205	PQ083840	Soil from banana field
TCS85	*T.* cf. *asperellum*	PQ555286	PQ569842	PQ569840	Soil from banana field
R39	*T. koningiopsis*	MW579418	PQ062198	PQ083833	Roots of healthy sisal
R62	*T. spirale*	MW579419	PQ062199	PQ083834	Roots of healthy sisal
R72	*T. harzianum* (species complex)	MW579420	PQ062200	PQ083835	Roots of healthy sisal
R74	*T. harzianum* (species complex)	MW579421	PQ062201	PQ083836	Roots of healthy sisal
R75	*T. saturnisporum*	MW579422	PQ062202	PQ083837	Roots of healthy sisal
R78	*T. koningiopsis*	MW579423	PQ062203	PQ083838	Roots of healthy sisal
C153	*T. harzianum* (species complex)	MW579417	PQ062195	PQ058662	Stem of healthy sisal
F12	*T.* cf. *asperellum*	MW579416	PQ062196	PQ058663	Leaves of healthy sisal
F130	*T. lentiforme*	MW579418	PQ062197	PQ058664	Leaves of healthy sisal

## Data Availability

All data generated or analyzed during this study are included in this published article and the Supplementary Materials.

## Supplementary Materials

The following supporting information can be downloaded at: https://www.mdpi.com/article/10.3390/jof10120860/s1, Figure S1. Top image: CaM-based ML phylogeny; Middle image: Tef-based ML phylogeny; Bottom image: ITS-based ML phylogeny.
