# Supplementary material for: Native and Non-Native Soil and Endophytic Trichoderma spp. from Semi-Arid Sisal Fields of Brazil Are Potential Biocontrol Agents for Sisal Bole Rot Disease
Source: J Fungi (Basel). 2024 Dec 11;10(12):860. doi: 10.3390/jof10120860 (PMC11678323; doi:10.3390/jof10120860)
Supplement: Supplementary file 1 [file jof-10-00860-s001.zip › jof-3169712-supplementary.pdf]

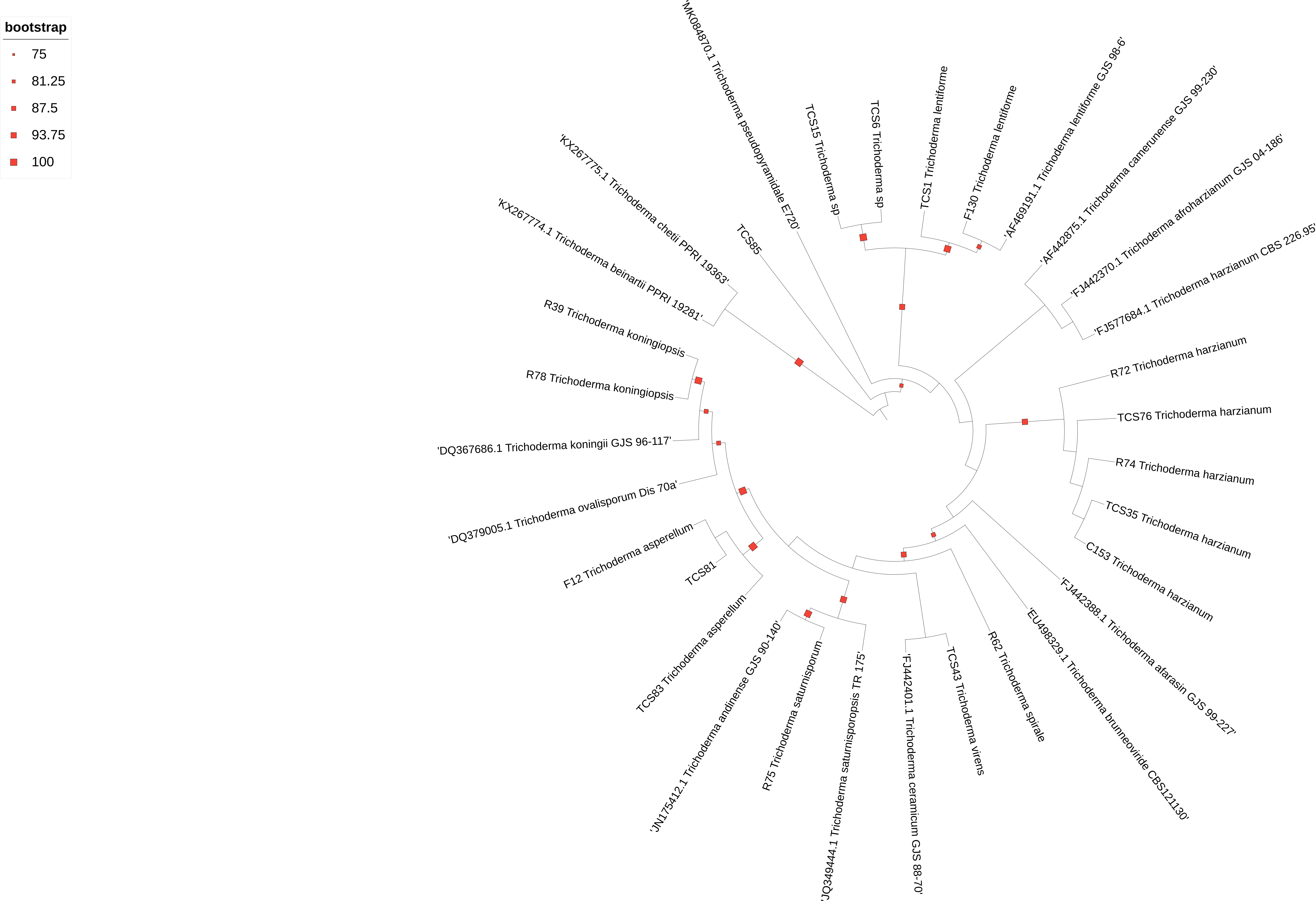
bootstrap
75
81.25
87.5
93.75
100
'MK084870.1 Trichoderma pseudopyramidale E720'
TCS15 Trichoderma sp
TCS6 Trichoderma sp
TCS1 Trichoderma lentiforme
F130 Trichoderma lentiforme
'AF469191.1 Trichoderma lentiforme GJS 98-6'
'AF442875.1 Trichoderma camerunense GJS 99-230'
'FJ442370.1 Trichoderma afroharzianum GJS 04-186'
'FJ577684.1 Trichoderma harzianum CBS 226.95'
R72 Trichoderma harzianum
TCS76 Trichoderma harzianum
R74 Trichoderma harzianum
TCS35 Trichoderma harzianum
C153 Trichoderma harzianum
'FJ442388.1 Trichoderma afarasin GJS 99-227'
'EU498329.1 Trichoderma brunneoviride CBS121130'
R62 Trichoderma spirale
TCS43 Trichoderma virens
'FJ442401.1 Trichoderma ceramicum GJS 88-70'
'JQ349444.1 Trichoderma saturnisporopsis TR 175'
R75 Trichoderma saturnisporum
'JN175412.1 Trichoderma andinense GJS 90-140'
TCS83 Trichoderma asperellum
TCS81
F12 Trichoderma asperellum
'DQ379005.1 Trichoderma ovalisporum Dis 70a'
'DQ367686.1 Trichoderma koningii GJS 96-117'
R78 Trichoderma koningiopsis
R39 Trichoderma koningiopsis
'KX267774.1 Trichoderma beinartii PPRI 19281'
'KX267775.1 Trichoderma chetii PPRI 19363'
TCS85

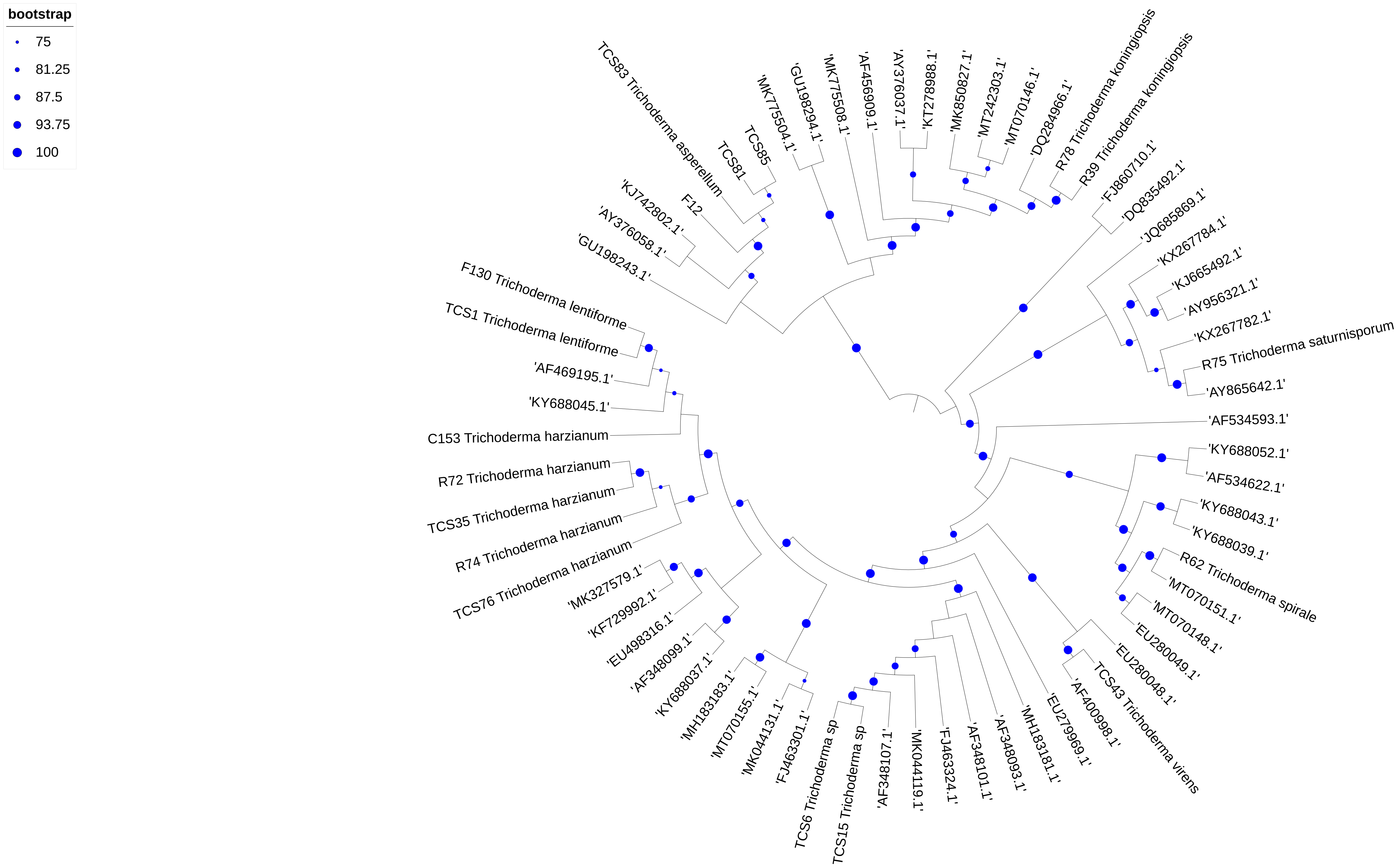
bootstrap
75
81.25
87.5
93.75
100
TCS83 Trichoderma asperellum
TCS81
TCS85
'MK775504.1'
'GU198294.1'
'MK775508.1'
'AF456909.1'
'AY376037.1'
'KT278988.1'
'MK850827.1'
'MT242303.1'
'MT070146.1'
'DQ284966.1'
R78 Trichoderma koningiopsis
R39 Trichoderma koningiopsis
'FJ860710.1'
'DQ835492.1'
'JQ685869.1'
'KX267784.1'
'KJ665492.1'
'AY956321.1'
'KX267782.1'
R75 Trichoderma saturnisporum
'AY865642.1'
'AF534593.1'
'KY688052.1'
'AF534622.1'
'KY688043.1'
'KY688039.1'
R62 Trichoderma spirale
'MT070151.1'
'MT070148.1'
'EU280049.1'
'EU280048.1'
TCS43 Trichoderma virens
'AF400998.1'
'EU279969.1'
'MH183181.1'
'AF348093.1'
'AF348101.1'
'FJ463324.1'
'MK044119.1'
'AF348107.1'
TCS15 Trichoderma sp
TCS6 Trichoderma sp
'FJ463301.1'
'MK044131.1'
'MT070155.1'
'MH183183.1'
'KY688037.1'
'AF348099.1'
'EU498316.1'
'KF729992.1'
'MK327579.1'
TCS76 Trichoderma harzianum
R74 Trichoderma harzianum
TCS35 Trichoderma harzianum
R72 Trichoderma harzianum
C153 Trichoderma harzianum
'KY688045.1'
'AF469195.1'
TCS1 Trichoderma lentiforme
F130 Trichoderma lentiforme
'GU198243.1'
'AY376058.1'
'KJ742802.1'
F12

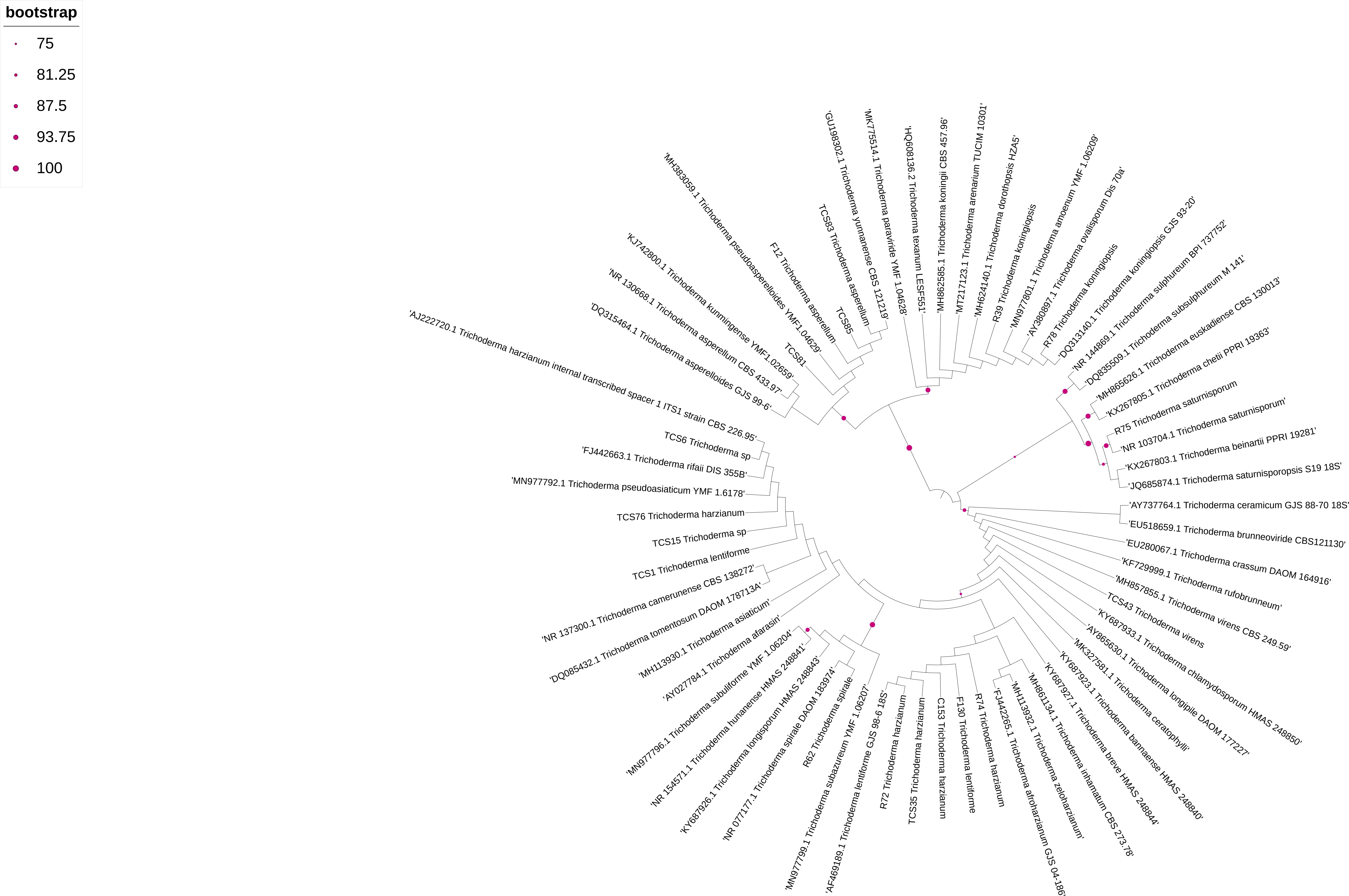
bootstrap
75
81.25
87.5
93.75
100
'AJ222720.1 Trichoderma harzianum internal transcribed spacer 1 ITS1 strain CBS 226.95'
'DQ315464.1 Trichoderma asperelloides GJS 99-6'
'NR 130668.1 Trichoderma asperellum CBS 433.97'
'KJ742800.1 Trichoderma kunmingense YMF1.02659'
'MH383059.1 Trichoderma pseudoasperelloides YMF1.04629'
TCS81
F12 Trichoderma asperellum
TCS85
TCS83 Trichoderma asperellum
'GU198302.1 Trichoderma yunnanense CBS 121219'
'MK775514.1 Trichoderma paraviride YMF 1.04628'
'HQ608136.2 Trichoderma texanum LESF551'
'MH862585.1 Trichoderma koningii CBS 457.96'
'MT217123.1 Trichoderma arenarium TUCIM 10301'
'MH624140.1 Trichoderma dorothopsis HZA5'
R39 Trichoderma koningiopsis
'MN977801.1 Trichoderma amoenum YMF 1.06209'
'AY380897.1 Trichoderma ovalisporum Dis 70a'
R78 Trichoderma koningiopsis
'DQ313140.1 Trichoderma koningiopsis GJS 93-20'
'NR 144869.1 Trichoderma sulphureum BPI 737752'
'DQ835509.1 Trichoderma subsulphureum M 141'
'MH865626.1 Trichoderma euskadiense CBS 130013'
'KX267805.1 Trichoderma chetii PPRI 19363'
R75 Trichoderma saturnisporum
'NR 103704.1 Trichoderma saturnisporum'
'KX267803.1 Trichoderma beinartii PPRI 19281'
'JQ685874.1 Trichoderma saturnisporopsis S19 18S'
'AY737764.1 Trichoderma ceramicum GJS 88-70 18S'
'EU518659.1 Trichoderma brunneoviride CBS121130'
'EU280067.1 Trichoderma crassum DAOM 164916'
'KF729999.1 Trichoderma rufobrunneum'
'MH857855.1 Trichoderma virens CBS 249.59'
TCS43 Trichoderma virens
'KY687933.1 Trichoderma chlamydosporum HMAS 248850'
'AY865630.1 Trichoderma longipile DAOM 177227'
'MK327581.1 Trichoderma ceratophylli'
'KY687923.1 Trichoderma bannaense HMAS 248840'
'KY687927.1 Trichoderma breve HMAS 248844'
'MH861134.1 Trichoderma inhamatum CBS 273.78'
'MH113932.1 Trichoderma zeloharzianum'
'FJ442265.1 Trichoderma afroharzianum GJS 04-186'
R74 Trichoderma harzianum
F130 Trichoderma lentiforme
C153 Trichoderma harzianum
TCS35 Trichoderma harzianum
R72 Trichoderma harzianum
'AF469189.1 Trichoderma lentiforme GJS 98-6 18S'
'MN977799.1 Trichoderma subazureum YMF 1.06207'
R62 Trichoderma spirale
'NR 077177.1 Trichoderma spirale DAOM 183974'
'KY687926.1 Trichoderma longisporum HMAS 248843'
'NR 154571.1 Trichoderma hunanense HMAS 248841'
'MN977796.1 Trichoderma subuliforme YMF 1.06204'
'AY027784.1 Trichoderma afarasin'
'MH113930.1 Trichoderma asiaticum'
'DQ085432.1 Trichoderma tomentosum DAOM 178713A'
'NR 137300.1 Trichoderma camerunense CBS 138272'
TCS1 Trichoderma lentiforme
TCS15 Trichoderma sp
TCS76 Trichoderma harzianum
'MN977792.1 Trichoderma pseudoasiaticum YMF 1.6178'
'FJ442663.1 Trichoderma rifaii DIS 355B'
TCS6 Trichoderma sp

Supplementary Material: Top image: CaM-based ML phylogeny; Middle image: Tef-based ML phylogeny; Bottom image:ITS-based ML phylogeny.
